# Genetic Variations in the Purinergic P2X7 Receptor Are Associated with the Immune Response to Ocular Toxoplasmosis in Colombia

**DOI:** 10.3390/microorganisms11102508

**Published:** 2023-10-07

**Authors:** Carlos A. Naranjo-Galvis, Rima McLeod, Jorge Enrique Gómez-Marín, Alejandra de-la-Torre, Cristian Rocha-Roa, Néstor Cardona, Juan Carlos Sepúlveda-Arias

**Affiliations:** 1 Facultad de Salud, Universidad Autónoma de Manizales, Manizales 170002, Colombia; 2 Department of Ophthalmology and Visual Sciences and Pediatrics (Infectious Diseases), The University of Chicago, Chicago, IL 60637, USA; 3 Grupo GEPAMOL, Centro de Investigaciones Biomédicas, Universidad del Quindío, Armenia 630001, Colombia; 4 Grupo de Investigación en Neurociencias (NeURos), Neurovitae Research Center, Escuela de Medicina y Ciencias de la Salud, Universidad del Rosario, Bogotá 110111, Colombia; 5 Facultad de Odontología, Universidad Antonio Nariño, Armenia 630004, Colombia; 6 Grupo Infección e Inmunidad, Facultad de Ciencias de la Salud, Universidad Tecnológica de Pereira, Pereira 660003, Colombia

**Keywords:** ocular toxoplasmosis, *P2RX7*, polymorphisms, protein structural modeling

## Abstract

Ocular toxoplasmosis (OT) is characterized by inflammation within the eye and is the most recognized clinical manifestation of toxoplasmosis. The objective of this study was to identify new single-nucleotide polymorphisms (SNPs) in the *P2RX7* gene that may have significance in the immune response to OT in Colombian patients. A case–control study was conducted to investigate the associations between SNPs (rs1718119 and rs2230912) in the *P2RX7* gene and OT in 64 Colombian patients with OT and 64 controls. Capillary electrophoresis was used to analyze the amplification products, and in silico algorithms were employed to predict deleterious SNPs. Stability analysis of amino acid changes indicated that both mutations could lead to decreased protein structure stability. A nonsynonymous SNP, Gln460Arg, located in the long cytoplasmic tail of the receptor, showed a significant association with OT (Bonferroni correction (BONF) = 0.029; odds ratio OR = 3.46; confidence interval CI: 1.05 to 11.39), while no significant association between rs1718119 and OT risk was observed. Based on the 3D structure analysis of the *P2RX7* protein trimer, it is hypothesized that an increase in the flexibility of the cytoplasmic domain of this receptor could alter its function. This SNP could potentially serve as a biomarker for identifying Colombian patients at risk of OT.

## 1. Introduction

Toxoplasmosis is a disease caused by the widely prevalent parasite *Toxoplasma gondii* [1]. This parasite has the ability to infect approximately one-third of the global human population, putting everyone at risk of infection. However, only a limited number of individuals experience symptoms [2,3]. In countries such as Colombia, Brazil, and the United States, it has been estimated that approximately 10–20% of individuals infected with *T. gondii* after birth develop ocular lesions. This suggests that the variability in clinical manifestations may be influenced by factors such as differences in parasite strains (isolates) and host susceptibility [4,5,6,7,8,9].

The purinergic P2X7 receptor (*P2RX7*) is highly expressed by cells of the hematopoietic lineage and, in the brain, is a member of the P2X subfamily activated by extracellular ATP [10]. ATP allows the flow of potassium and sodium, triggers innate immune responses, can mediate the killing of infectious microorganisms, supports the establishment of long-lived memory CD8^+^T cells [11], and stimulates inflammatory processes [12]. Purinergic receptors are found on all types of cells in mammalian tissues; *P2RX7* KO mice showed increased susceptibility to toxoplasmosis [13], with tissue damage characterized by the impaired production of pro-inflammatory cytokines (IL-12, IL-1β, IFN-γ, and TNF-α) [14]. Recently, the P2X7 receptor has been implicated in the immune response to *T. gondii*. P2X7 receptor-mediated killing of *T. gondii* is associated with phagolysosome formation that is not related to the generation of nitric oxide and is accompanied by host-cell apoptosis and the production of free oxygen radicals [15,16].

Therefore, we aimed to investigate the C-terminal region of this gene in ocular toxoplasmosis by studying possibly functional, nonsynonymous polymorphisms within a ~7.2 kb long region around Gln460Arg, the most widely investigated SNP in the *P2RX7* gene, including the Ala348Thr (rs1718119) variant. The functional polymorphism rs2230912 (Gln460Arg) is located in exon 13 of the *P2RX7* gene and is believed to have implications for cytolysis/apoptosis, intracellular signaling, and changes in cell morphology [17]. It is likely to be involved in protein–protein interactions that affect *P2RX7*-mediated signaling [18].

*P2RX7* has been associated with susceptibility to toxoplasmosis in the United States, Europe, and Brazil [19]. However, there is a lack of data regarding the significance of polymorphisms within this gene in determining susceptibility to the development of OT in Colombian patients. Therefore, we conducted a case–control study involving Colombian patients with OT.

## 2. Materials and Methods

### 2.1. Patients and Healthy Subjects

We conducted this study from January 2017 to June 2019. The sample size was calculated taking into account the frequency of the −592 A polymorphism of the IL-10 gene in the healthy population of Medellín (Colombia), which had a value of 0.28. n = Z^2^pq/d^2^. A total of 128 participants were included in this case-control study; 64 cases (34 men and 30 women) of IgG (+) with OT and 64 healthy individuals (28 men and 36 women) of IgG (−) were diagnosed as previously described [7,20]. The Ethics Committee of the Universidad Tecnológica de Pereira approved this study (Act 06-2012).

### 2.2. Genomic DNA Isolation

Genomic DNA was extracted from blood samples using a QIAamp DNA Blood Mini kit (Qiagen, Hilden, Germany), following the manufacturer’s protocols. Serum samples were also analyzed for anti-*Toxoplasma* IgG and IgM antibody titers using a commercial ELISA assay, as per the manufacturer’s instructions (ELFA commercial, Kit VIDAS TOXO IgG II Ref. 30210, TOXO IgM Ref. 30202 BioMérieux CIBM).

### 2.3. Genotyping of P2RX7 (rs1718119 and rs2230912) SNPs

We identified coding single-nucleotide polymorphisms (SNPs) for the *P2RX7* gene using the SNP database of the National Center for Biotechnology Information (http://www.ncbi.nlm.nih.gov/SNP, accessed on 15 May 2023). Specifically, we selected two nonsynonymous SNPs: rs1718119 (Ala348Thr) and rs2230912 (Gln460Arg). SNPs located in the coding regions of genes are more likely to result in functional differences compared with those found elsewhere [21]. The amplification products were subsequently analyzed using capillary electrophoresis. We employed the mini-sequencing technique, also known as “ddNTP primer extension”, as previously described [7].

To genotype the rs1718119 A/G, and the rs2230912 C/T polymorphisms in the *P2RX7* gene, we performed multiplex PCR amplification in a Veriti thermal cycler (Applied Biosystems, Foster City, CA, USA). The final reaction volume was 10 µL, containing 1 to 10 ng of genomic DNA, 1 µL of Qiagen Multiplex PCR master mix (Qiagen, Hilden, Germany), and 0.2 to 0.6 µM of the following primers: for rs1718119, forward primer (F) 5′-TGCAGCTTGAAGCAAAAGAG-3′, reverse primer (R) 5′-TGTCGATGAGGAAGTCGATG-3′ and extension probe used 5′-CTCTCTCTCTCTCTCTCTCTCTCTCTCTCTTTGCATTC-3′; for rs2230912, forward primer (F) 5′-TTCCTGGACAACCAGAGGAG-3′, reverse primer (R) 5′-TCCTGGTAGAGCAGGAGGAA-3′ and extension probe used 5′-CTCTCTCTCTCTCTCTCTCTCTCTCTCTCTCTCTAGTCGCCTCCTTTTAAGCAGC-3′. The amplification conditions consisted of an initial denaturation step of 95 °C for 10 min, followed by 35 cycles of 95 °C for 1 min, 60 °C for 90 s, and 72 °C for 50 s. Finally, there was a 10 min extension step at 72 °C.

### 2.4. In Silico Functional Analysis

The full amino acid sequence of *P2RX7* was retrieved from the National Center for Biotechnology Information (https://www.ncbi.nlm.nih.gov/protein/, accessed on 15 May 2023) with the accession number Q99572 for further analysis. Since P2RX7 functions as a trimer, we focused on the sequence representing one of its three chains for this particular study.

The regulatory potential of candidate SNPs associated with OT was assessed through in silico analysis using RegulomeDB (Version 1.1), available at (http://regulome.stanford.edu/, accessed on 15 May 2023) [22]. RegulomeDB (https://www.regulomedb.org, accessed on 15 May 2023) is a comprehensive database that annotates the SNPs within the intergenic regions of the Homo sapiens genome, providing information on known regulatory DNA elements such as transcription factor binding sites, promoter regions, and DNAase hypersensitivity regions. The scoring system employed by RegulomeDB ranges from 1 to 7, with a score of 7 indicating a lack of available data on the SNP’s functional impact. Scores 4, 5, or 6 suggest minimal evidence of binding effects on transcription factors (TFs). Scores 3a and 3b indicate a lower likelihood of affecting binding, while scores 2a to 2c imply that the SNP is expected to impact binding. Scores 1a–1f indicate that the SNP is likely to affect the expression of a specific target gene.

The prediction of deleterious SNPs was extended by using a mixture of numerous in silico algorithms, including Expasy ProtParam (https://web.expasy.org/protparam/, accessed on 15 May 2023) [23] to predict physicochemical parameters of the P2X7 protein; SIFT [24], Polyphen-2 [25], and PROVEAN [26] to predict functionally important nsSNPs; SNPs & GO [27] to predict disease-associated nsSNPs; and TMHMM [28], Protter [29], and SOPMA [30] to predict transmembrane domains of P2X7 and secondary structure. The I-Mutant2.0 [31] online server was used to predict the effect of the mutations found under selective pressure on protein stability.

In order to elucidate the impact of the Gln460Arg mutation on the protein structure and its potential influence on functionality, we generated a three-dimensional model of the trimer using the crystal structure with code PDB 6U9W [32] as a template. The Swiss-model web tool was utilized for this prediction. The stereochemical quality of each chain in the final model was assessed using a Ramachandran plot, obtained through the MolProbity web tool [33]. All visualizations were conducted using UCSF Chimera software (Chimera-1.17.2) [34].

### 2.5. Expression QTLs

To evaluate the effect of candidate SNPs on gene expression levels (cis- and trans-eQTL) for ocular toxoplasmosis, data from the Blood eQTL browser (http://genenetwork.nl/bloodeqtlbrowser/, accessed on 15 May 2023) and Consortium for the Architecture of Gene Expression (CAGE) (http://cnsgenomics.com/shiny/CAGE/, accessed on 15 May 2023) [35] were obtained. The significance of eQTLs was determined using a false discovery rate (FDR) ≤ 0.05.

### 2.6. Statistical Analysis

At each SNP, allelic association was assessed between cases and controls using the X^2^ test with 1 d.f. Allelic odds ratios (ORs) and 95% confidence intervals (95% CIs) were calculated using PLINK software (version 4.2) [36]. Hardy–Weinberg (HW) equilibrium was separately tested for all polymorphisms between cases and controls using the X^2^ test. The Haploview v4.1 program (www.broad.mit.edu/mpg/haploview, accessed on 15 May 2023) was employed to calculate the linkage disequilibrium (LD) structure among SNPs in *P2RX7* and the haplotype association approach. The Bonferroni correction was applied, and significance was established at a *p* value of <0.05.

## 3. Results

### 3.1. Association between P2RX7 Polymorphisms and OT Susceptibility

In this study, a total of 64 cases and 64 controls were included for evaluation. The genotypes for rs1718119 and rs2230912 were found to exhibit no significant deviation from the Hardy–Weinberg equilibrium. To investigate the potential independent risk factor of the less-represented alleles of SNPs in the candidate gene (*P2RX7*) for toxoplasmosis, an association test was conducted (Table 1).

In our study population, a significant association with OT was observed for the rs2230912 polymorphism in the *P2RX7* gene (BONF = 0.029; OR = 3.46; 95% CI: 1.05 to 11.39). However, no association was found between the observed and expected distributions of the rs1718119 allele in the *P2RX7* gene within this population. Furthermore, there were no significant differences in the allelic frequencies of these polymorphisms between patients with OT and control subjects (rs1718119 (BONF = 0.55)).

**Figure 1:**
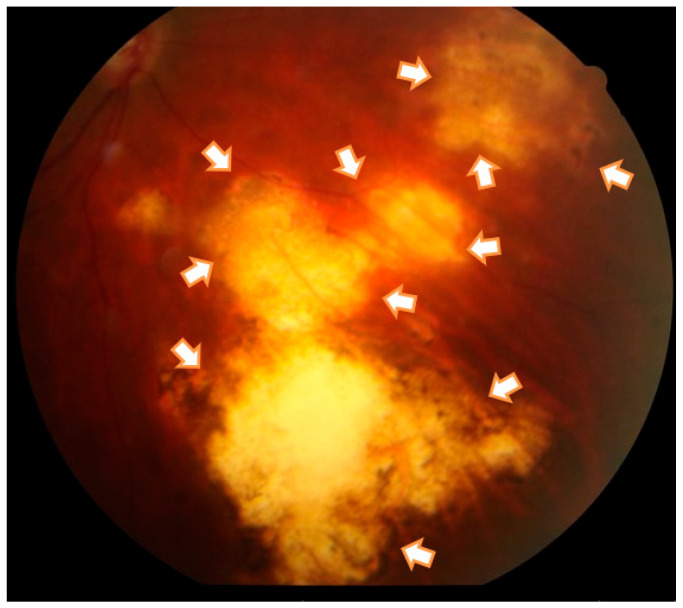
Right eye fundus photography from a Colombian patient with multiple and extensive chorioretinal scars (white arrows). This patient showed IL-10 levels overpassing IFN-γ production (IL-10/IFN-γ ratio of 1:3), indicating a Th2 skewed response during ex vivo stimulation of polymorphic mononuclear cells, as described in another study [37].

The haplotype frequencies of *P2RX7* (rs1718119 and rs2230912) were analyzed, and their association with OT was evaluated. Three haplotype combinations were observed: G/A, A/A, and A/G (*p* = 0.73, *p* = 0.48, and *p* = 0.035). The only significant difference in haplotype frequencies between cases and healthy controls was found for the A/G haplotype. Among the controls, the A allele was present in 124 out of 128 individuals (96.7%), while the G allele was present in only 4 out of 128 individuals (3.1%). In contrast, among OT cases, the A allele was found in 25 out of 128 individuals (19.5%), and the G allele was found in 103 out of 128 individuals (80.4%) (OR = 3.61; CI: 1.11 to 11.75). As an example of cases associated with this allele and exhibiting extensive lesions, a photograph of the retina of a patient with OT carrying the G allele is provided (Figure 1).

### 3.2. In Silico Analysis

The *P2RX7* protein consists of 595 amino acid residues with a molecular weight of 68,584.93 Da. The theoretical pI was 8.57; likewise, the aliphatic index and grand average of hydropathicity were 77.11 and −0.303, respectively. The instability index (II) was computed to be 46.36, and Expasy ProtParam classified the protein as unstable.

Although SNPs do not have a regulatory mechanism as predicted with RegulomeDB, polymorphisms rs1718119 and rs2230912 are known to be involved in proximal regulation and may influence post-transcriptional regulation [22]. The score of “1” for rs2230912 indicates that it may affect binding and is linked to the expression of a gene target (eQTL + TF binding/DNase peak); a score of “4” for the polymorphism rs1718119 indicates that there is minimal binding evidence (TF binding + DNase peak). Table 2 presents data showing the prediction of functional consequences of nsSNPs in the *P2RX7* gene using PROVEAN, SIFT, and Polyphen-2.

Two nsSNPs (rs11718119 (disease probability: 0.013) and rs2230912 (disease probability: 0.035)) analyzed by the SNP & GO tool were predicted to be neutral (scores < 0.5). The results of the TMHMM server analysis predict that the P2RX7 protein has a transmembrane domain. The compositions of the secondary structure of the P2RX7 protein were as follows: 20.8% extended strand, 27.39% alpha-helix, 47.73% random coil, and 4.03% beta-turn.

The I-Mutant tool analyzed the effect of variants rs11718119 and 2230912 on protein stability. According to our results, both nsSNPs (rs1718119: −2.15 Kcal/mol; rs2230912: −1.59 Kcal/mol) cause an increase in energy levels, so they are predicted to decrease the stability of protein structure.

**Figure 2:**
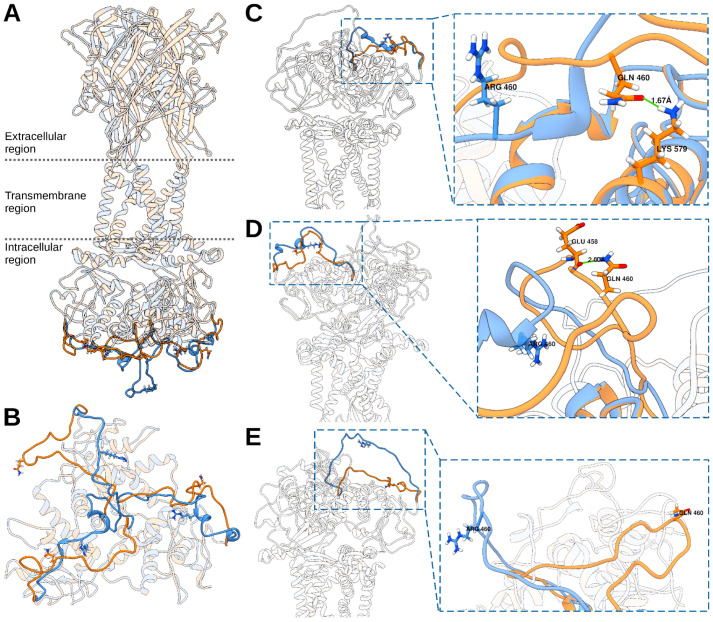
Predicted Tertiary Structures of Wild-Type and Mutant *P2RX7* from the online server Swissmodel (https://swissmodel.expasy.org/, accessed on 15 May 2023). (**A**) Structural alignment of the two complexes: the wild type and the mutant are depicted in light orange and light blue, respectively. The extracellular, transmembrane, and intracellular regions are displayed for visual reference. (**A**,**B**) The primary structural difference between the two forms is highlighted in a solid color, representing the loop where the Q460R residue is situated. (**C**–**E**) Key interactions (depicted as solid green lines) involving the Q460 residue in Chains A (**C**), B (**D**), and C (**E**). In all cases, the wild-type and mutant structures are shown in orange and blue, respectively.

**Figure 3:**
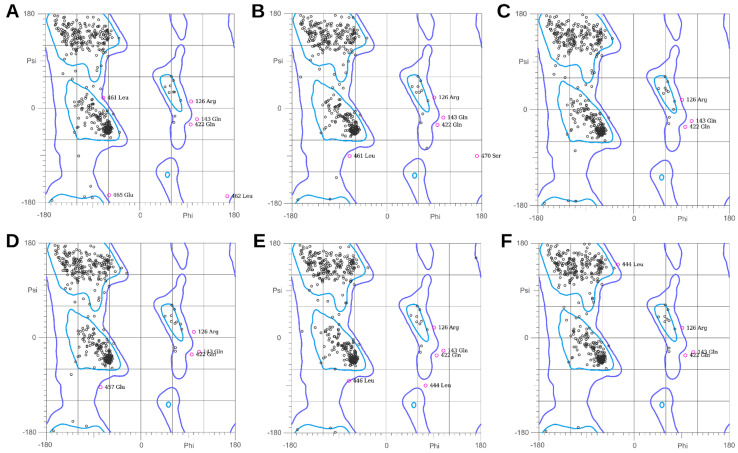
Assessment of the three-dimensional structure of constructed models using Ramachandran plots for the chains A (**A**), B (**B**), and C (**C**) of *P2RX7*–WD and the chains A (**D**), B (**E**), and C (**F**) of *P2RX7*–QR. All the chains presented percentages greater than 98% of residues in allowed regions.

The sequence template from Rattus norvegicus and the human P2RX7 sequence showed an identity percentage of 80.34% and a coverage percentage of 100%. The whole structure, both the wild-type version (*P2RX7*-WD) and the mutated (*P2RX7*-QR), is shown aligned in Figure 2A. A Ramachandran plot was used to predict the probability of particular amino acids forming secondary structures based on the dihedral angles, namely, Ψ and ɸ, of amino acids, which are calculated based on the Van der Waals radius of side-chain atoms. The evaluation of the stereochemical quality of the constructed models suggests that, in both cases, the three protein chains have percentages of amino acids in allowed regions greater than 98% (Figure 3).

On the other hand, based on our protein structural modeling results, it was observed that only the change in Asn460Arg induces a noticeable change in the loop that connects the amino acid Leu442-Ser474. In the case of *P2RX7*-QR, this loop is more exposed to the solvent; this loop is highlighted in Figure 2B. From visual inspection, we identified that changing a polar amino acid (Gln) for a positively charged amino acid (Arg) in the A chain (Figure 2C) causes a hydrogen bond between Gln460 and Lys579 to be lost. In *P2RX7*-QR, Arg460 is expected to generate electrostatic repulsion with the positively charged side chain of Lys579, facilitating Leu442-Ser474 loop displacement. In chain B (Figure 2D), it was observed that Gln460 presented a hydrogen bond with the backbone carbonyl of the nearby residue Glu458, and, when mutated by a more voluminous residue (Arg), it is expected that a steric hindrance occurs, similar to what was observed in the A chain, which facilitates the displacement of the Leu442-Ser474 loop. For the C chain (Figure 2E), no interactions were observed for Gln460. However, the change in Arg markedly displaces the loop, similar to the A and B chains. This suggests that the Gln460Arg mutation induces the loss of strong interactions in *P2RX7*-WD that modify the structure of the cytoplasmic domain.

It has been reported that the absence of the cytosolic domain does not alter the sensitivity of the P2X7 receptor for ATP and therefore does not alter its functionality [32]. However, changes in the cytosolic domain can alter the location of the receptor on the plasma membrane [38] or even cause an imbalance of zinc ions, which can modulate cellular processes such as apoptosis [39]. These imbalances can favor modulation of the host-cell response by *T. gondii*, which can modulate the apoptotic responses of host cells to survive in infected cells [40]. Based on the above and taking into account that (i) the conformation of the Leu442-Ser474 loop changes markedly for *P2RX7*-QR concerning *P2RX7*-WD and that (ii) the zinc binding sites (one on each chain of the trimer) are localized just at the end of these loops, we hypothesize that the Gln460Arg mutation can trigger changes in the cytosolic domain that alter the binding of zinc ions or even substrates such as GDP. Therefore, for further studies, it would be interesting to address problems such as the influence of the Leu442-Ser474 loop on the binding of zinc atoms to the cytoplasmic domain. One of the alternatives is molecular dynamics simulations, which can help clarify the role of mutations on proteins, as they have been applied in, to mention some examples, inherited bleeding disorder [41], bacterial drug resistance [42], protein folding in prion diseases [43], and others. Furthermore, it is of utmost importance to gather experimental data from wet laboratory experiments to effectively complement the conducted structural analysis in order to validate the interpretations derived from our computational models.

### 3.3. Expression QTLs

Among the studied SNPs, rs2230912 affects transcription of the genes *CAMKK2* (*p* ≤ 6.7 × 10^−21^, Pfdr = 0), *COQ5* (*p* ≤ 5.4 × 10^−13^, pFDR = 0), *P2RX7* (*p* ≤ 5.4 × 10^−10^, pFDR = 0), and *C12orf43* (*p* ≤ 3.2 × 10^−5^, pFDR < 0.01) in peripheral blood (cis-eQTL). No statistically significant trans-eQTL effects were detected (pFDR ≤ 0.05).

## 4. Discussion

Certain genotypes have been observed to make individuals more susceptible to toxoplasmosis, potentially indicating an increased risk of acquiring the infection or a greater severity of the disease [44]. Genetic polymorphisms can lead to changes in the expression or function of the molecules they encode [45]. In this study, we conducted a case−control association study using a candidate gene approach to investigate the potential association between genetic variants (rs1718119 and rs2230912) in the *P2RX7* gene and an increased susceptibility to OT within the Colombian population. A total of 64 Colombian OT patients (cases) and 64 healthy controls were included in the study for further analysis of the association between *P2RX7* SNPs (rs1718119 and rs2230912) and OT.

The present study provides compelling evidence linking the nonsynonymous single-nucleotide polymorphism (SNP) *P2RX7*-Gln460Arg, situated in the long cytoplasmic tail of the receptor, to a significant reduction in normal receptor function among Colombian patients. This association is further supported by in silico predictions. Remarkably, the frequency of this polymorphism is notably higher in patients with OT. The impact of this polymorphism may be attributed to its effect on the binding of the C-terminal domain of *P2RX7* to various intracellular signaling components, particularly in individuals with OT who are homozygous for the variant. Model predictions indicate that this alteration is likely to affect *P2RX7* dimerization and protein–protein interactions [46]. The susceptibility mutation in P2RX7 (rs2230912) described in this study corresponds to a missense mutation leading to the amino acid change from Gln460 to Arg. This residue is located within an SH3-like domain of the C-terminal region, which is conserved across rodents and humans. It plays a crucial role in important protein functions such as intracellular signaling, the formation of large pores, receptor trafficking, and membrane blebbing [47].

Two studies have demonstrated that ATP binding to *P2RX7* triggers the fusion of lysosomes with the parasitophorous vacuole, resulting in the elimination of *T. gondii* tachyzoites in both murine and human macrophages [15,16]. Interestingly, a gain-of-function single-nucleotide polymorphism (SNP) in *P2RX7* has been associated with resistance to both congenital and OT [19]. While further research is necessary to establish the causal role of the Gln460Arg mutation, it has been identified as a genetic variant that increases susceptibility to OT.

The identification of P2 receptors involved in the proinflammatory effects of extracellular nucleotides, particularly ATP, holds significance in purinergic signaling and may pave the way for the development of novel anti-inflammatory drugs. *P2RX7*, in particular, has been extensively studied in relation to its role in the inflammatory response [48]. Over the past decades, research has unveiled the pivotal involvement of the *P2RX7*-Gln460Arg receptor in a wide range of pathological and physiological processes, including severe sepsis [49], osteoporosis [50], bipolar disorder, major depressive disorder [51,52], and infections [53].

The substitution of the Gln460Arg amino acid in *P2RX7* is believed to have an impact on signal transduction, potentially through its interaction with various components of intracellular signaling. This single-nucleotide polymorphism (SNP) is located in the death domain (~residues 430–530), which belongs to a class of protein motifs known as the death fold. The death domain facilitates protein oligomerization and is present in numerous proteins involved in apoptosis and inflammation. Within this domain, there is a proline-rich region (~residues 450–456) that serves as the binding site for the human SH3 domain, which spans approximately 60 amino acids and participates in various intracellular signaling pathways. Interaction with this SH3 binding domain, encompassing the Gln460Arg polymorphism, ultimately leads to the phosphorylation of ERK 1/2. Therefore, the hetero-oligomerization involving *P2RX7*-Gln460Arg can alter the conformation of the domain responsible for interacting with Src tyrosine kinases, resulting in modified and reduced ERK 1/2 signal transduction [54,55]. These findings lend support to the hypothesis that the *P2RX7* signaling pathways play a role in OT.

*P2RX7*, previously recognized for its cytotoxic activity, is an ATP-gated, non-selective cation channel that belongs to the ionotropic P2X receptor family. It is also known as one of the “death receptors” and plays a crucial role in regulating programmed cell death in various pathologies [56]. *T. gondii*, a parasite with immune modulation capabilities, including the ability to manipulate apoptosis, downregulates the apoptotic response of host cells, as demonstrated in murine models and human cells [40]. The parasite achieves this by manipulating signaling pathways to suppress immune responses, thereby promoting its own survival [57]. During invasion and while residing within the parasitophorous vacuole, *T. gondii* releases proteins from dense granules (GRAs), micronemes (MICs), and rhoptries (ROPs). These parasite-derived proteins have been implicated in apoptosis modulation [40]. For instance, GRA5 has been associated with the inhibition of apoptosis in infected cells [58], while GRA3 may act as a regulator of the intrinsic pathway of apoptosis by inhibiting proapoptotic proteins. The modulation of immune system responses by the parasite could serve as a feedback mechanism that potentially exacerbates cellular damage in individuals with the reported polymorphism. However, further experiments are required to validate this hypothesis.

Gene coexpression networks serve multiple purposes, such as functional gene annotation, regulatory gene identification, and prioritization of candidate disease genes [59]. In our analysis, we observed that the *P2RX7* gene exhibits coexpression with genes that possess expression quantitative trait loci (eQTLs) specific to monocytes or CD4+ T cells. This finding carries significant implications for the identification of the risk factors associated with *T. gondii* infection.

The utilization of association analysis and in silico coexpression analysis alone does not provide conclusive evidence to determine whether the disequilibrium in allele distribution is responsible for pathological changes or if it is merely a result of haplotype inheritance. In a recent study, we conducted an analysis of the effect of *P2RX7* alleles on cytokine levels in a group of patients with ocular toxoplasmosis [60]. In contrast to the current findings, which demonstrate a disequilibrium in the distribution of the rs2230912 allele of the *P2RX7* gene between cases with OT and those without, the observed difference was associated with a change in the G nucleotide of the rs1718119 allele. Within the Hernandez-de-los-Rios study, we performed a functional analysis of cytokine levels specifically in cases with ocular toxoplasmosis, and it was found that the A/A and G/A alleles resulted in significantly higher levels of IL-1β compared with the G/G genotype [57]. No significant changes in cytokine levels were observed for the rs2230912 allele of the *P2RX7* gene upon ex vivo induction with live tachyzoites, both in nonocular cases and within cases with ocular toxoplasmosis, when considering clinical variables such as the number of lesions or the level of inflammation [60]. The functional analysis of the association between other polymorphisms, expression quantitative trait loci (eQTLs), for the *CAMKK2*, *COQ5*, *P2RX7*, and *C12orf43* genes in relation to the single-nucleotide polymorphism rs2230912, remains to be conducted to assess its effect on cytokine response in ex vivo models of infection [61].

The observed disequilibrium in the distribution of the rs2230912 allele between individuals with and without ocular toxoplasmosis, along with the significant differences in IL-1β levels in patients with ocular toxoplasmosis and the rs1718119 alleles of the *P2RX7* gene as previously described [60], suggests a linkage between this gene and the pathology of ocular toxoplasmosis. However, further detailed analysis is required to understand how nucleotide sequence changes affect the immune cytokine network response. It is now known that IL-10 levels are increased in ocular toxoplasmosis, while IL-1β is associated with more severe lesions [62]. While parasite virulence factors play a crucial role in the primary immune response, another factor is involved during reactivation episodes in the secondary immune response [60]. Future studies should investigate how allelic variation influences the modification of cytokine responses in greater detail.

## 5. Conclusions

In summary, our findings indicate a significant association between OT and a nonsynonymous SNP, Gln460Arg, located in the long cytoplasmic tail of the *P2RX7* receptor. This SNP holds potential as a biomarker for identifying Colombian patients at risk of developing OT. These results provide support for the involvement of *P2RX7* signaling pathways in the pathogenesis of OT. In future studies, it may be useful for devising diagnostic and therapeutic approaches in OT to determine whether these polymorphisms alter cytokine production in patients with ocular compromise and if they influence the clinical presentation of disease.

## Figures and Tables

**Table 1:** Association analysis of MAF alleles for SNPs in *P2RX7* gene.

Polymorphisms	Location	X^2^	OR	95% CI	BONF
1718119	Exon 11	0.23	1.55	[0.71–3.35]	0.55
**2230912**	**Exon 13**	**3.62**	**3.46**	**[1.05–11.39]**	**0.029**

X^2^: chi-square; OR: odds ratio; 95% CIs, 95% confidence intervals for odds ratio; BONF: Bonferroni correction.

**Table 2:** Prediction of functionally important consequences of nsSNPs in the *P2RX7* gene.

SNP ID	Location	Position	Amino Acid Change	PROVEAN Score	SIFT Score	Polyphen-2 Score
*rs1718119*	Exon 11	120099486	A348T	3.136 Neutral	0.95 Tolerated	0.000 Benign
*rs2230912*	Exon 13	120106579	Q460R	−0.855 Neutral	0.14 Tolerated	0.005 Probably damaging effect

PROVEAN score: ≤2.5 predicted as deleterious variations; SIFT score: ≤0.05 predicted to be deleterious, >0.05 expected to be tolerated; Polyphen-2 score: ranges from 0 to 1 (0 indicates a benign effect).

## Data Availability

Not applicable.
